# Interfacial Debonding Detection for Rectangular CFST Using the MASW Method and Its Physical Mechanism Analysis at the Meso-Level

**DOI:** 10.3390/s19122778

**Published:** 2019-06-20

**Authors:** Hongbing Chen, Bin Xu, Jiang Wang, Lele Luan, Tianmin Zhou, Xin Nie, Yi-Lung Mo

**Affiliations:** 1 Department of Civil Engineering, Tsinghua University, Beijing 100084, China; hongbingchen2019@163.com; 2 College of Civil Engineering, Huaqiao University, Xiamen 361021, China; 1611404010@hqu.edu.cn; 3 Fujian Provincial Key Lab of Intelligent Infrastructures and Monitoring, Huaqiao University, Xiamen 361021, China; 4 Department of Civil and Environmental Engineering, Northeastern University, Boston, MA 02115, USA; luan.l@husky.neu.edu; 5 Department of Civil and Environmental Engineering, University of Houston, Houston, TX 77204-4006, USA; zhoutm2011@gmail.com (T.Z.); yilungmo@egr.uh.edu (Y.-L.M.)

**Keywords:** concrete-filled steel tube (CFST), multichannel analysis of surface wave (MASW), interfacial debonding detection, nondestructive testing (NDT), finite element analysis, mesoscale analysis

## Abstract

In this study, the transient multichannel analysis of surface waves (MASW) is proposed to detect the existence, the location and the length of interface debonding defects in rectangular concrete-filled steel tubes (CFST). Mesoscale numerical analysis is performed to validate the feasibility of MASW-based interfacial debonding detection. Research findings indicate that the coaxial characteristics in the Rayleigh wave disperse at the starting point of the debonding area and gradually restores at the end of the defect. For healthy specimens, the surface wave mode in CFST is closer to the Rayleigh wave. However, it can be treated as a Lamb wave since the steel plate is boundary-free on both sides in the debonding area. The displacement curves are further investigated with forward analysis to obtain the dispersion curves. The mesoscale numerical simulation results indicate that the propagation characteristic of the surface wave is dominated by the debonding defect. The detectability of interfacial debonding detection for rectangular CFST using the MASW approach is numerically verified in this study. The proposed MASW-based nondestructive testing technique can achieve bond-slip detection by comparing the variation trend of the coaxial characteristics in the time-history output signals and the dispersion curves obtained from the forward analysis, for avoiding misjudgment of the experimental observations.

## 1. Introduction

### 1.1. Necessity of Interface Debonding Detection for CFSTs 

Due to the distinct advantages of mechanical behavior and construction convenience, the concrete-filled steel tubes (CFSTs) has become the primary load-bearing components of large-scale civil infrastructures, including ultra-high-rise buildings, long-span bridges, and harbor projects [1,2]. As the continuously increased demand in load-bearing capacity and the complicated internal structure of the CFST components, together with the influence of unscientific construction scheme, the temperature variation and the effect of the live load during the service period, the interfacial debonding and local cavities are easily formed, especially in CFST with giant cross-sections [3]. Taking Tianjin Goldin Finance 117 Tower as an example [4], the geometrical area of the cross-section is even up to 45 m^2^. The construction quality of large-scale composite components and the effective NDT testing and real-time monitoring of defects insides steel-concrete composites, such as interfacial debonding and bond-slip defects, have become research hotspots in engineering and academia. However, the interfacial bonding condition is vitally significant to guarantee the interoperability between concrete core and steel tubes.

### 1.2. Various NDT Detection Techniques for Interfacial Debonding Defects

Generally, the interface problem extensively exists in steel reinforced concrete composite structures, such as the bond-slip between reinforcing bars and concrete [5,6,7,8]. The bonding condition of the cohesive layer between concrete core and the carbon fiber reinforced plastics (CFRP) is also a critical concern in multi-layered composite materials. Recently, interfacial debonding detection techniques have been proposed and developed by using bulk wave and guided wave methods for the structural health monitoring of CFSTs, tubes and pipes, and multi-layered plate-like structures [9,10,11]. In CFST structures, the Young’s modulus and density of steel plates are obviously higher than that of the concrete core, resulting in significant deviation in wave propagation characteristics. Hence, the conventional ultrasonic method, acoustic emission technology, and other traditional non-destructive approaches [12,13,14,15] are ineffective to detect the interfacial debonding behind the steel plate. The electromagnetic wave was validated as an efficient NDT method in the debonding detection between CFRP and concrete [16]. However, the shielding effect of steel media makes electromagnetic waves unable to penetrate through the steel tubes. The piezoelectric lead-zirconate-titanate (PZT) patches are widely employed as actuators and sensors in the damage evaluation in timber [17], steel reinforced concrete structures, and concrete-encased composite structures [18] subjected to the shear/flexural monotonic and cyclic loading [19,20,21,22,23,24,25] and the interfacial debonding detection for CFSTs with different geometries [26,27]. For achieving the determination of the degree of composite action between concrete and metal, Giri et al. proposed a novel feature extraction method using piezoelectric transducers to predict the gaps between a steel plate and concrete. The research findings indicate that gaps smaller than 0.1 mm can be successfully detected [28]. Moreover, the shrinkage-induced delamination of ultra-high-performance concrete bonded over an existing concrete substrate has been detected using distributed fiber optic sensors [29].

To realize the debonding detection for the steel-concrete components, Xu et al. [30,31,32,33] developed the debonding detection system composed of surface-mounted PZT patches and embedded smart aggregates (SA) based on the wave propagation method and the impedance technique. To further uncover the detection mechanism of the PZT based SHM approach, the wave propagation process was investigated using numerical analysis modeled with homogenous concrete and numerical concrete sample at the mesoscale [34,35]. In addition, the multi-physical coupling analysis based on conventional finite element methods and spectral finite element methods (SFEM) [36] was introduced to study the propagation mechanism of stress wave in CFSTs and the impedance characteristic variation of PZT transducers, which significantly promoted the development of PZT based interfacial debonding detection for CFST structures. During the propagating process of stress wave excited by PZT actuator, additional reflection and diffraction wave will occur while encountering debonding defects, resulting in apparent attenuation of signal amplitude and wave energy [37,38,39]. In addition, the arriving time will be slightly postponed since the traveling path of the diffraction wave is commonly extended due to the existence of debonding defects [40]. Based on the comparative analysis of the received voltage signal, it is possible to evaluate the health status of the CFST component. In fact, the debonding detection based on transmission waves still belongs to the category of the bulk wave. However, the longitudinal wave, shear wave, and the surface wave account for 7%, 26%, and 67% of the total wave energy. Compared with the bulk waves, the wave energy of the surface wave is relatively higher, which makes it relatively easier to be identified in the time-history waveform [41,42]. 

### 1.3. Guided Wave-Based NDT Testing and Its Application in Debonding Detection

Due to the unique forming and propagating mechanism, the guided wave attenuates slowly and is especially suitable for the health monitoring and defect detection for long-distance structures [43,44,45,46]. Ordinary waveguides are slender rods and tubes, single or multi-layered plates [47]. Since the characteristics of the guided wave are mainly dependent on the geometrical shape while the material properties are identical, the symmetrical, antisymmetric, and axisymmetric modes are usually observed in different waveguides. Different from the bulk wave, the frequency-dependent characteristics, including multi-mode and dispersion phenomenon, make the wave propagation of the guided wave extremely complicated [48,49]. Hence, systematic investigation on the guided wave is a pre-requisite for choosing the appropriate excitation and mode type to improve the accuracy and efficiency of the NDT tests. The ultrasonic waves generated by the PZT patches, electro-acoustic transducer and lasers have been applied to the health monitoring of bond failure, the delamination in multi-layered plate-like composites, the debonding caused by corrosion of the steel bars and the defect in tubular waveguides [50,51,52,53,54,55,56,57,58]. The slender CFSTs specimens can be analyzed as rod-like waveguides since the diameter-height ratio is very small. However, the radius and height of CFSTs commonly adopted in current super high-rise builds are about 1.0 m and 4.0 m, respectively. Therefore, the CFST members are not suitable to be simplified as rod-like waveguides. Essentially speaking, the dispersion curve mainly depends on the geometric characteristics of the waveguide, thus deriving a variety of nondestructive testing methods based on the guided wave theory. For CFST members, the presence of concrete and its internal defects will affect the wave propagation behavior in steel plate. In addition, the corrosion defects in the steel plate also lead to the variation of the dispersion characteristics of the steel plate. Damage identification can be achieved by analyzing the difference in the time-history guided wave and the dispersion curves. The feasibility of the Lamb wave theory in the monitoring of debonding defects and steel plate corrosion has been experimentally verified with two oblique incidence sensors [59]. However, the proposed approach needs a water tank as the coupling agent, which limits the application of this method in practical engineering. Recently, the piezoelectric-based sensory technique using guided wave propagation has been introduced to monitor the gap in carbon fiber-reinforced polymer-strengthened concrete, which provides a promising and cost-effective solution for gap detection and concrete characterization [60]. The guided wave measurement is also applied in the debonding damage assessment in RC structures. The experimental findings matched well with that of numerical simulation constructed with the spectral finite element (SFE) method [61]. Giri et al. performed guided wave-based NDT testing on the debonding detection in a carbon fiber-reinforced concrete structure. It has been proved that the developed damage indices were sensitive to the extent of the debonding defect and correlated linearly with the debonding evolution [62]. 

### 1.4. MASW-Based Structural Health Monitoring for Composite Structures

The MASW method was initially proposed by Al-Husseini et al. [63,64,65], and this method was eventually popularized by Park et al. [66]. In recent years, the MASW method has become a mature technique in the geologic survey [67,68], and the corresponding technical regulation has also been issued [69]. The horizontal resolution of the MASW method on the variation of the propagation medium has also been numerically and experimentally validated by Mi et al. [70]. While changing the material properties of the bonding layer [71], the dispersion curves of the steel plate were investigated using the MASW method. The evaluation of the interfacial bonding status was achieved by observing the difference of the phase velocity between the dispersion curves obtained from the forward analysis on the time-history signals observed from each sensor and the theoretical dispersion curve of the Lamb wave. However, the variation of the bonding condition was simulated by adjusting the material properties of the whole cohesive layer. In general, the interfacial debonding defects are usually locally imperfect, which are entirely different from the case mimicked by globally weakening the mechanical strength of the bonding material. Moreover, the ideal Lamb wave only exists in the thin plate with infinite dimension. Compared with air or vacuum, the material difference between steel and concrete is relatively smaller. Additionally, the thickness of concrete is apparently larger than that of steel plate. It is reasonable to simplify the CFST as a semi-infinite concrete space covered with a thin steel plate. The ideal Rayleigh wave in the semi-infinite homogeneous space is dispersion-free. However, for layered medium composed of different materials, the Rayleigh waves will exhibit apparent multi-mode characteristics and obvious dispersion phenomenon. Due to the disappearance of lateral confinement provided by the concrete core from the beginning of the debonding defect, the steel plate turns to be the waveguide for the Lamb wave. However, with the recovery of lateral and vertical constraints, the CFST component is again close to the waveguide of the Rayleigh wave. The numerical simulation result indicates that the Rayleigh wave passing through the defect can be decomposed into a transmitted wave with a large wavelength and a short-wavelength waveform near the internal cracks or defects, which makes it easier to evaluate the defects’ dimension using appropriate filtering methods [72].

For bulk wave measurement-based debonding detection for CFSTs, the PZT-based functional smart aggregate(SA) has to be embedded in the concrete due to the significant attenuation of high-frequency stress wave in the concrete core [34], which is not suitable for completed structures. In current surface wave measurement-based NDT testing [73], the surface-mounted PZT sensors are arranged in a pitch-catch manner without pre-burying SAs. In this case, the detection precision is limited since it is difficult to take full advantage of the dispersion characteristics of the Lamb and Rayleigh waves propagating in CFST components without and with debonding defects. For avoiding the shortcomings of conventional bulk wave and surface wave-based NDT method using PZT sensors, a novel debonding detection method using MASW is proposed to evaluate the health status of CFST components. In order to improve the detection accuracy, the MASW method is introduced to analyze the influence of the interfacial debonding defect on the signal waveform of MASW measurement. Through the comparison of dispersion curves from theoretical analysis corresponding to Rayleigh or Lamb waves and that from the forward analysis, the wave attributes can be clearly identified. Thus, the existence, and even the specific location, of debonding defects can be determined accordingly. The research conclusions indicate that the proposed interfacial debonding detection approaches based on surface wave theory and the MASW method are feasible and practical, which provide a novel and effective solution for the debonding detection for CFST structures. The research findings in this paper can be directly used to optimize the sensor arrangement and signal selection, and lay a theoretical foundation for its practical application. 

## 2. MASW Method and F-K Transformation

**Figure 1:**
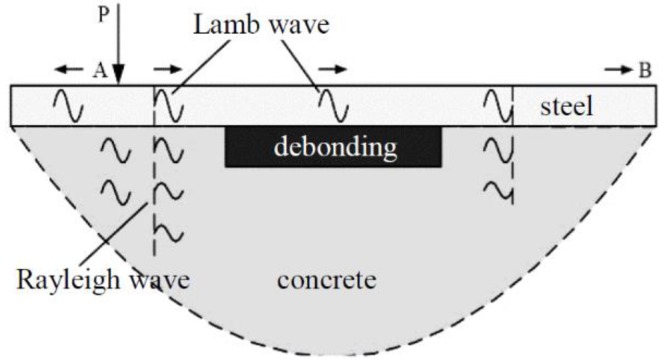
The surface wave propagation in CFSTs.

Aside from the shortcoming of the aforementioned debonding detection technique using bulk wave measurement, the PZT SAs have to be embedded in the concrete core due to the significant wave attenuation in the concrete core, especially for the CFST specimens with very large cross-sections. However, it is impossible to implant the SAs into CFSTs for completed structures. In this study, nondestructive testing for CFSTs based on the surface wave method is proposed and the wave propagation in the CFST can be succinctly described as shown in Figure 1.

**Figure 2:**
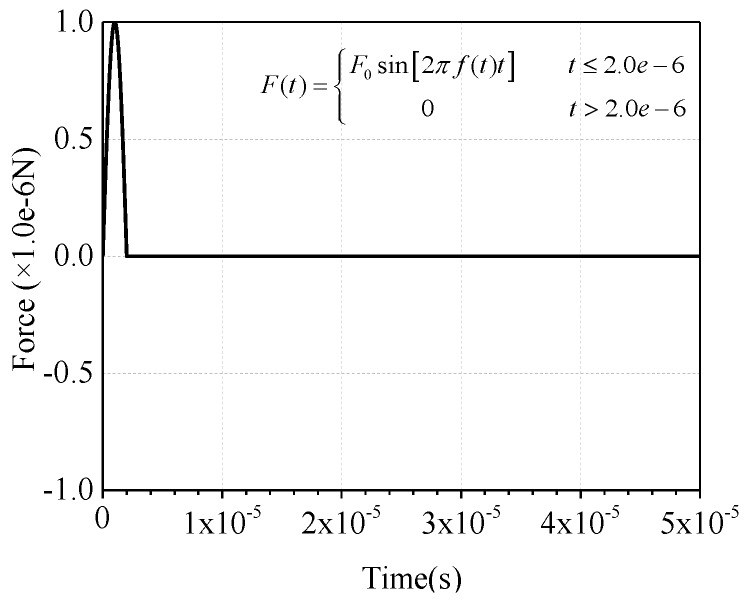
The half-period sine pulse signal.

**Figure 3:**
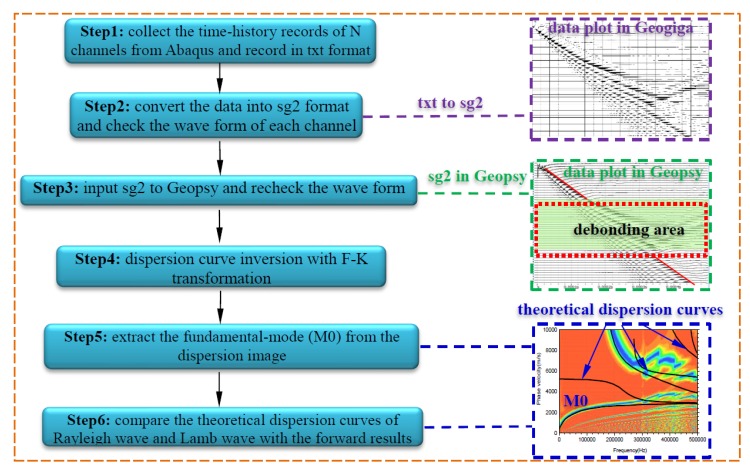
The data analysis process for the MASW method.

In the practical MASW test, the drop hammer is commonly adopted for excitation and the signal can be simplified as a half-period sine pulse signal as presented in Figure 2 [71]. Generally, the sensors are linearly arranged in the MASW test [74], and the dispersion curve can be derived through forwarding analysis on the output signal collected from each sensor using F-K transformation [74,75]. Therefore, the location of debonding defects can be determined by observing the variation of output data in the time domain. Furthermore, the stress wave attribute can be identified according to the comparison of dispersion curves from forwarding analysis and the theoretical calculation. The data analysis process for the MASW method is detailed in Figure 3.

As presented in Figure 3, the time-history measurement is recorded in txt format and then will be converted into the sg2 format by means of the software of Geogiga Front End 8.3 (Geogiga Technology Corp., Calgary, Alberta, Canada) [76]. Then, the time-history files in sg2 format will be input into Geopsy [75], which is also an open source software for geophysical research. With the help of Geopsy, the forwarding analysis with F-K transformation can be performed. The stress wave attributes can be determined by comparing the fundamental-mode (M_0_) from the dispersion image with the theoretical dispersion curves of the Rayleigh wave and Lamb wave. Basically, if the fundamental-mode (M_0_) matches well with the theoretical dispersion curves of the Rayleigh wave, the monitored specimen is healthy without interfacial debonding. Otherwise, the M_0_ will be close to the theoretical dispersion curves of the Lamb wave. The theoretical basis of MASW method and the dispersion analysis with F-K transformation have been reported in detail [74,75] and are excluded in this study.

## 3. Mesoscale Modeling and Wave Propagation Analysis

As a typical multi-phase composite material, the microstructure of the concrete core and its material strength in different local regions present strong randomness characteristics. In addition, the debonding defect in CFST belongs to local damage. Therefore, it is necessary to validate the reliability and stability of the proposed method through mesoscale FEM analysis, ensuring that the measurement is dominantly influenced by the debonding defect other than the mesostructure variation of the concrete core. In addition to the CFST models constructed with homogeneous concrete, the mesoscopic models are built using the random aggregate method in this section. 

### 3.1. Mesoscale Modeling of Concrete Based on RAM

**Figure 4:**
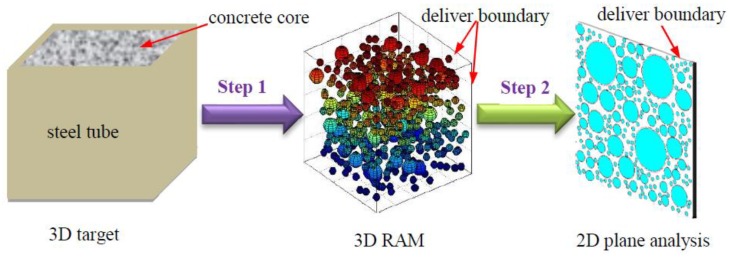
The procedures for simplifying the 3D models to the 2D plane analysis.

**Figure 5:**
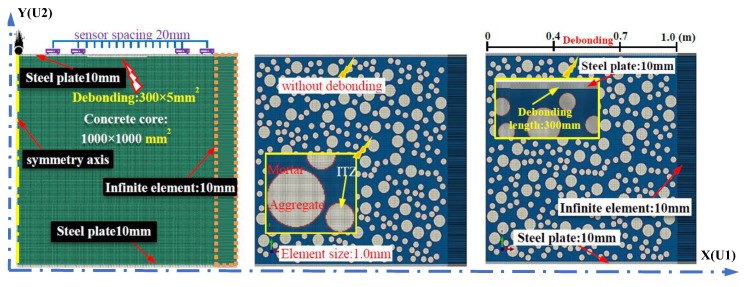
The element meshing and boundary setting (mesh size: 1.0 mm).

Based on the Fuller’s grading curve and the Walraven method [77,78], the 3D and 2D numerical concrete models can be established at the meso-level. The procedures for simplifying the 3D models to 2D planar analysis are shown in Figure 4. The circular aggregates are generated as per the numerical study performed by Xu et al. [34]. In order to simplify the numerical simulation, the CFST specimen is established as a two-dimensional model. For practical engineering structures, the diameter or side length of CFST component is about 1.0 m. Therefore, the dimension of the concrete core is set to 1.0 m × 1.0 m, and the thickness of steel plate takes the value of 10 mm. The width of the infinite element area shown in Figure 5 is set to 0.1 m to improve the absorption efficiency of stress wave. The absorption efficiency of the artificial damping boundary and the infinite element will be discussed in detail in the flowing Section 3.2. Basically, the integration step and the mesh size have to be strictly limited in wave propagation analysis to ensure the accuracy and smoothness of the waveform in the output signals. For the purpose of presenting a clear wave filed and considering the material variation at the interfaces between steel-mortar, mortar-ITZ, and ITZ-aggregates, the mesh size of 2D numerical models with and without debonding defects constructed with homogeneous material assumption and mesoscale numerical concrete core is set to 1.0 mm, which is determined by Equations (1) and (2) [31]. In this study, the exterior boundary of the concrete core with the dimension of 1.0 m × 1.0 m is treated as the delivering boundary of aggregates. The detailed information about the aggregate generation and packing program has been reported [79], which is omitted in this study.

(1) h≤λ/5

(2) $$\mathrm{dt}=\mathrm{CFL}\times \frac{h}{\min\left(C_{S},C_{P}\right)}=0.2\times \min\left(C_{S},C_{P}\right)+\frac{\mathrm{dt}}{h}=\min\left(C_{S},C_{P}\right)/25$$

where h and λ are the maximum element size and wavelength. C_S_, T_S_, C_P_, and T_P_ correspond to the velocities and time period of the shear wave and longitudinal wave, respectively.

In this study, the debonding defects with different lengths in CFSTs are simulated by deleting the corresponding elements in the FEM models [34]. The element meshing and boundary setting in the FEM models of CFST members are exhibited in Figure 5. The material parameters of the concrete, aggregate, the interfacial transition zone (ITZ), the mortar and steel plate are identical to that of the numerical study performed by Xu et al. [34]. The elastic material properties of the concrete core and steel tubular are tabulated in Table 1.

### 3.2. Boundary Condition

**Figure 6:**
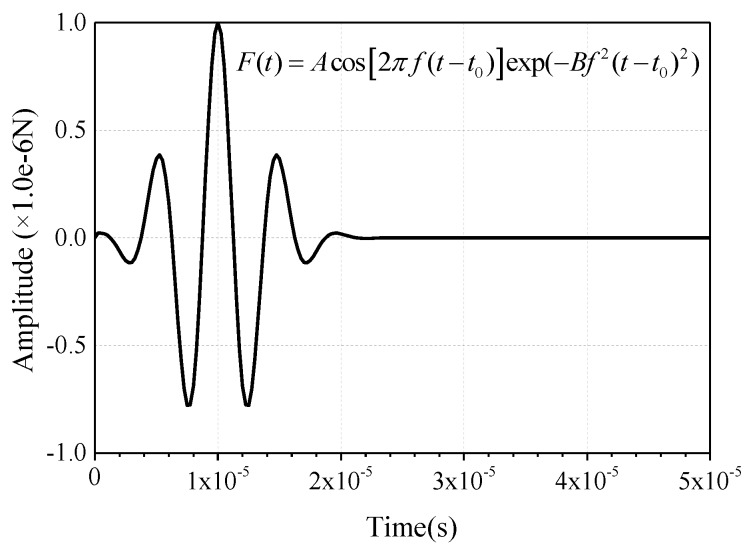
The cosine wavelet voltage signals (200 kHz).

**Figure 7:**
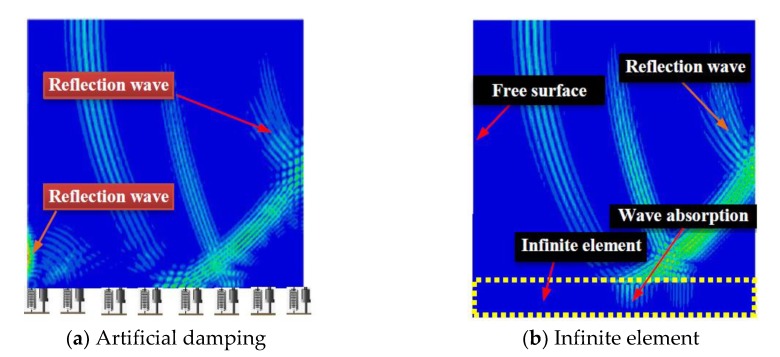
Comparison of absorption efficiency of different boundaries.

Since the geometrical dimension of CFST is much larger than the wavelength of the ultrasonic wave, the borderline of the propagation medium is usually regarded as a free boundary in the time-history analysis of stress wave propagation. In order to reduce the computational cost, a symmetrical boundary is adopted at the excitation point, as presented in Figure 5. As mentioned above, the height of the CFST is greater than its cross-sectional width, so the absorption boundary is required in the height direction of the numerical models. The frequently-used absorption boundary in Abaqus is the artificial damping element or the infinite element [80,81]. In order to realize the wave energy concentration and enhance the contrast effect, the cosine wavelet signal is employed as the excitation herein. Compared with other wavelets, the cosine wavelet is more suitable for the excitation and simulation of surface wave [82]. The mathematical expression and the function image are shown in Figure 6. Here, the t_0_ is set to 1.0 × 10^−5^ s. The absorption effect corresponding to these two methods on the stress wave is shown in Figure 7. It can be seen that the absorption efficiency of the stress wave by the infinite element is slightly higher than that of the artificial damping boundary, without an additional reflection wave reverberating from the bottom boundary. Therefore, the numerical models in the following sections are modeled with the infinite element to lower the influence of the reflection wave caused by the geometry borderline in the FEM models.

### 3.3. Time-History Analysis of Stress Wave Propagation in Homogeneous and Mesoscopic Models with and without Debonding Defects

As discussed above, the excitation signal of the drop hammer can be simplified as a half-period sine pulse signal [71] in the practical MASW tests. In order to investigate the wave propagation excited by drop hammer, the signal amplitude of impulse force excitation is set to 1.0 × 10^−6^ N and the time duration takes the value of 2.0 × 10^−6^ s. 

**Figure 8:**
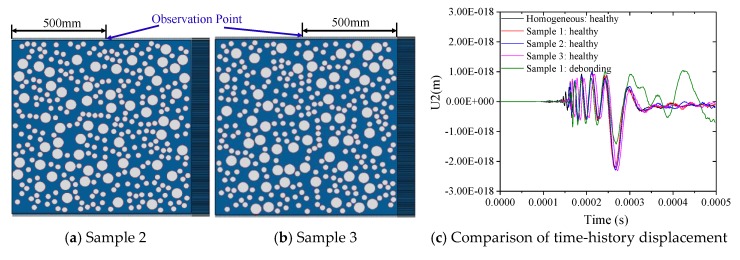
Influence of mesoscale structure variation on the propagation process of the surface wave.

Current numerical findings indicate that the mesoscale structure variation induced by the random aggregate samples with different geometries and distribution patterns poses an obvious influence on the NDT signals of bulk wave measurement while the surface-mounted PZT patch and the embedded smart aggregates (SA) are employed as actuators and sensors, respectively [34]. However, mesoscopic numerical simulation on the surface wave measurement-based debonding detection for rectangular CFST using the surface-mounted PZT as actuator and sensor indicates that the distribution variation of aggregates leads to a neglectable influence on the surface wave propagation while the surface-mounted PZT sensors are arranged in a pitch-and-catch manner [73]. In order to compare the influence of the aggregate distribution and debonding defects on the MASW measurement, two additional mesoscale CFST components as presented in Figure 8a,b are established and the time-history displacement in the vertical direction are illustrated in Figure 8c. It can be clearly observed from Figure 8c that the distribution variation of circular aggregates in the concrete core of the CFSTs poses very limited influence on the vertical displacement of the observation points, compared with that induced by the debonding defects.

As is known, the concrete is composed of aggregates, mortar, and the interfacial transition zone (ITZ) at the mesoscale. Therefore, the mesoscale simulation on the stress wave propagation in CFST with the dimension of 1.0 m × 1.0 m is extremely time-consuming since the element size is set to 1.0 mm and the element number is up to 1,000,000. It takes about 77.42–86.62 h to run each model. In order to balance the computational efficiency and analysis accuracy, the equivalent homogenization method-based multiscale finite element modeling approach will be introduced to improve the computational efficiency for stress wave propagation analysis and to further investigate the influence of geometry distribution variation of aggregate on the wave fields in CFSTs.

**Figure 9:**
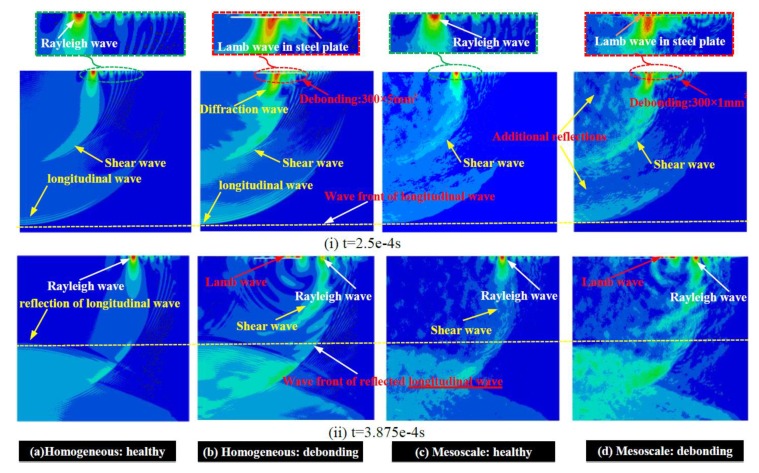
Wave fields comparison between the mesoscopic and homogenized models.

The comparison of the wave fields in numerical models of CFSTs established with a homogeneous core and mesoscale numerical concrete samples with and without considering the debonding defect are presented in Figure 9. At the time instant of 2.5 × 10^−4^ s, the waveforms of the shear wave, longitudinal wave, Rayleigh wave, and the Lamb wave in the cross-section of rectangular CFSTs are clearly visible. The wave velocity of the longitudinal wave is much higher than that of the other three. However, the wave amplitude of the longitudinal wave is the lowest. The peak value of waveform locates in the surface wave, followed by the shear wave. The distribution pattern of wave energy matches well with the research finding in the collected literatures [41,42]. 

Since the debonding defect is pre-set at the middle-upper part of the CFST and the impulse excitation is applied at the upper-left corner, the waveform of the longitudinal wave is slightly affected by the interfacial debonding defects, as presented in Figure 9a,b. Compared with the wave fields presented in Figure 9a, the longitudinal wave and shear wave in Figure 9b exhibit stronger waveforms, which implies that the wave energy distribution is slightly influenced by the debonding defects. However, the wave fields in Figure 9a,c are similar and additional refection can be clearly visible due to the material difference between aggregates and the ITZ, the ITZ and the mortar. Similar phenomenon can be observed by comparing Figure 9b,d. However, as exhibited in Figure 9b,d, the wave properties of surface wave are significantly changed when the surface wave is reaching the debonding defect. The surface wave will be divided into a Lamb wave propagating in the steel plate and the diffraction wave spreading in the concrete core. However, it can be observed that the surface wave energy is still concentrated in the surface wave crest at the time instant of 3.875 × 10^−4^ s. Meanwhile, the wave energy has been scattered by the debonding defects, resulting in the Lamb wave propagating in the steel plate above the interfacial defects. Moreover, a visible Rayleigh wave can be observed in damaged specimens after the stress wave passing through the debonding area. Comparatively, the debonding defect plays a leading role in the wave propagation of the surface wave. The wave propagation analysis indicates that the debonding detection based on surface wave theory is feasible, which also lays the foundation for performing the nondestructive testing for CFST components with the MASW method.

## 4. Dispersion Analysis Based on the MASW Method

In general, if the material properties between the monitored specimen and its subbase are quite different, the guided wave can be simplified as a Lamb wave. Hence, the bonding condition monitoring of the cohesive layer, the material property prediction and even the damage level evaluation can be carried out by comparing the variation of the dispersion characteristics [67,71,83]. However, for CFSTs, this kind of simplification is not rigorous enough since the elastic material strength between concrete and steel is relatively close, compared with the idealized waveguide for Lamb wave. Here, in order to figure out the wave attributes and mode components of stress wave in CFST specimens with and without debonding defects, the transient MASW method is employed for the dispersion analysis.

### 4.1. Theoretical Dispersion Curves of Rayleigh and Lamb Waves

**Figure 10:**
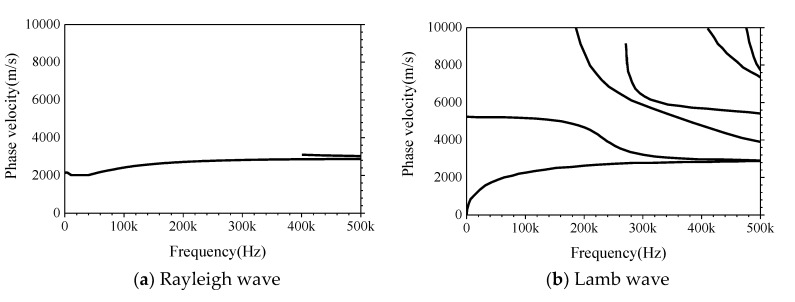
The theoretical dispersion curves of (**a**) Rayleigh and (**b**) Lamb waves.

The formation mechanisms of Lamb and Rayleigh waves are totally different. The ideal waveguide for the Lamb wave is an infinitely thin plate. In addition to the typical dispersion characteristics, the Lamb wave is usually multi-mode, which can also lead to complex mode transitions near the defects or boundaries [54,67,71,83,84,85,86]. The Rayleigh wave in a half-infinite homogeneous medium is dispersion-free. However, for multi-layered media composed of different materials, significant dispersion and multimode characteristics emerge during the propagation process of the Rayleigh wave [41,68,72,87,88]. The theoretical dispersion curves of the Lamb and Rayleigh waves corresponding to different medium assumptions are shown in Figure 10a,b. Here, the 2D CFST specimen is simplified as a two-layer planer for the theoretical dispersion analysis of the Rayleigh wave. Meanwhile, the steel plate with the thickness of 10 mm is considered as the waveguide of the Lamb wave. The theoretical dispersion curves are presented in Figure 10a,b.

Figure 10 indicates that the dispersion characteristics of the ideal Rayleigh and Lamb waves are remarkably different. The phase velocity of the Rayleigh wave is higher than that of the Lamb wave when excitation frequency is at a lower range. Moreover, the quantity of vibration modes of the Lamb wave is much larger than that of the Rayleigh wave. On the basis of the distinguishable difference between dispersion curves of the Lamb wave and Rayleigh wave, it is possible to classify the wave attributes of surface wave propagating in CFSTs with and without debonding defects by comparing the measured M_0_ curve form forward analysis and the fundamental mode M_0_s presented in Figure 10a,b.

### 4.2. Time-History Comparison of MASW Measurement

**Figure 11:**
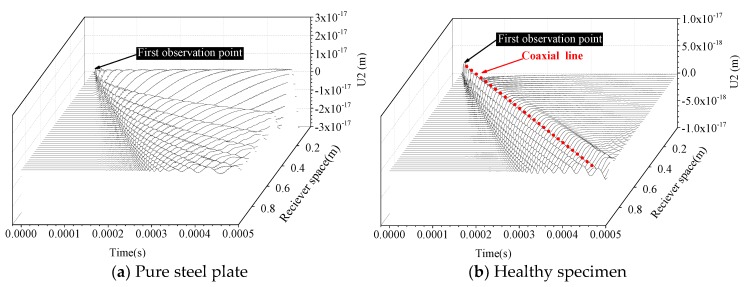

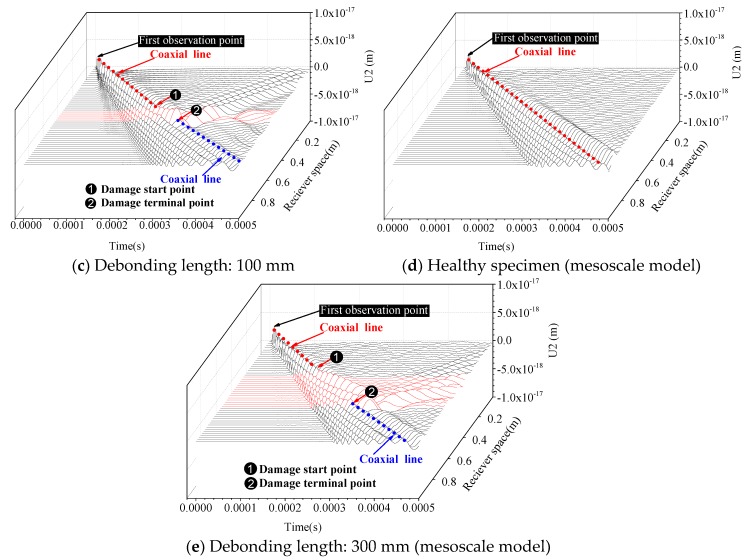
Time history displacement curve obtained with the MASW method.

According to the basic principle of MASW and the modeling approach mentioned above, the time-history curves of displacement in the vertical direction corresponding to the pure steel plate and CFST components with and without debonding defects are shown in Figure 11. In Figure 11, the *X*-axis represents the time series in seconds. In this study, the time-history displacement curves obtained with the MASW method in the duration of 5 × 10^−4^ s are selected for parametric study. The *Y*-axis titled as U2 denotes the specific displacement in the vertical direction, as presented in the Figure 5. The time-history curves corresponding to each channel are arranged parallel to the *Z*-axis. The specific value of the *Z*-axis stands for the distance between the observation points in MASW measurement and the excitation point.

As the thin steel plate is the waveguide for Lamb wave, the waveform presented in Figure 11a should belong to Lamb wave. It can be seen that the coaxial line, which is a typical characteristic of the Rayleigh wave, is clearly visible in Figure 11b. As shown in Figure 11c, the remarkable coaxial characteristic of the Rayleigh wave will disappear when debonding occurs, especially at the debonding area. The wave propagation patterns are Rayleigh wave before surface wave encountering and after passing through the interface debonding defect. However, the wavelength and signal amplitude suddenly increase at the top of debonding area. Similar phenomenon can be observed in Figure 11d,e, where the concrete cores are modeled with randomly-distributed circular aggregates. It can be concluded that the starting position and end point of debonding defects are easily determined by observing the time-domain variation of signal collected from MASW observations, providing a convenient and effective approach for debonding localization analysis.

### 4.3. The Dispersion Analysis with F-K Transformation

**Figure 12:**
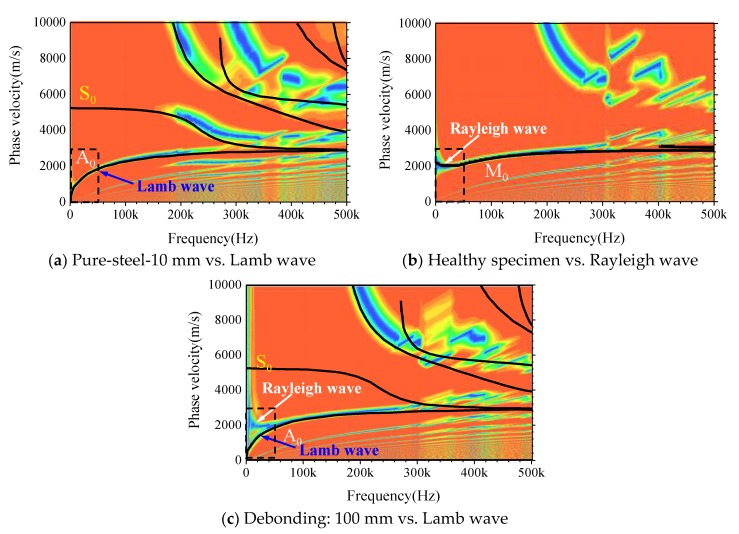
The comparison of dispersion curves from the forward analysis and theoretical calculation.

In order to determine the attributes of the stress wave in CFST components excited by the transient MASW method and validate the conclusions aforementioned, the time-history displacement curves derived from numerical models are further analyzed with F-K transformation. Furthermore, the dispersion figure can be obtained through the forward analysis. First of all, taking the pure steel plate with the thickness of 10 mm as an example, the feasibility and validity of the forward analysis on the MASW data shown in Figure 11a are verified by comparing the dispersion curves with that from theoretical analysis result presented in Figure 10. It can be seen from Figure 12a that the fundamental mode M_0_ in the dispersion figure matches well with that from the theoretical analysis of the Lamb wave. Moreover, the second modes are also in good agreement. The discrepancies still exist due to the fact that the wave absorption efficiency of the infinite element is limited. Moreover, the accuracy of forward analysis is also related to the position selected for data collection and the frequency of transient excitation. Nevertheless, the accuracy of the forward results in this paper is high enough to satisfy the precision requirement for general engineering application. Figure 12b is the dispersion figure corresponding to the MASW data presented in Figure 11b and the M_0_ agrees well with that of the theoretical dispersion curve of the Rayleigh wave. However, the M_0_ shown in Figure 12c turns to be like that the A_0_ mode of the Lamb wave when debonding occurs. As highlighted with the black dotted bordered rectangle at the lower-left corner of Figure 12a–c, the dispersion image measured with the MASW method presents as the combination of the dispersion characteristic of Lamb and Rayleigh waves when debonding occurs. Compared with Figure 12a,b the dispersion image in Figure 12c exhibits two branches when the frequency is lower than 50 kHz. More specifically, the lower branch matches well with the A_0_ mode of the Lamb wave and the upper branch is in good agreement with the M_0_ of the Rayleigh wave. In this study, the frequency of the half-period sine pulse signal in Figure 2 is set to 250 kHz. As presented in Figure 12, the phase velocity of A_0_ and S_0_ mode of the Lamb wave gradually converge to that of the M_0_ mode of the Rayleigh wave. Moreover, the Rayleigh wave significantly attenuates along the vertical direction of the propagation medium and low-frequency signals with relatively longer wavelength can penetrate the steel plate with larger thickness more easily. Meanwhile, high-frequency stress wave is more sensitive to the debonding defects. The relationship between the excitation signal frequency, the thickness of the steel plate and the dimension of the debonding defects have to be systematically investigated by means of experimental and theoretical analysis in further studies.

**Figure 13:**
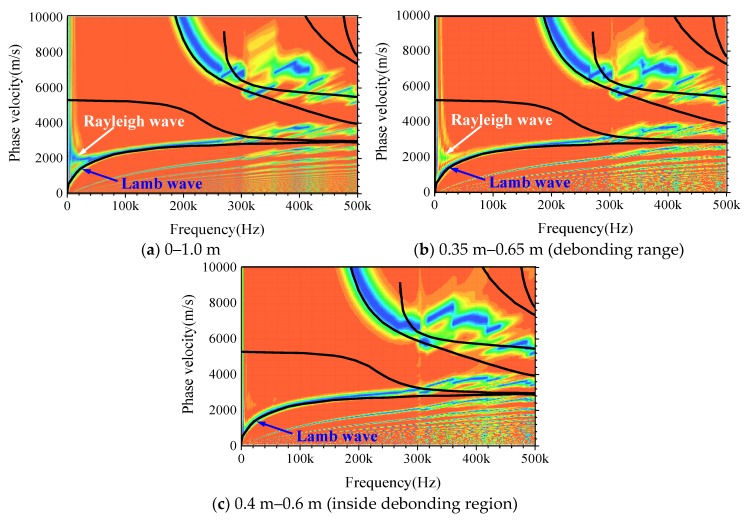
The comparison of dispersion curves with different measuring intervals.

The wave propagation analysis conducted in Section 3.3 indicates that the propagation characteristics of stress waves in CFST are greatly changed due to the appearance of debonding defect, making the surface wave in the healthy specimen vibrate in the hybrid mode of Rayleigh and Lamb waves. Moreover, the variation of M_0_ is significant, which provides a reliable criterion for debonding prediction. To improve the detection accuracy, three measuring interval length: 0–1.0 m, 0.35–0.65 m (debonding range) and 0.4–0.6 m (inside the debonding region), are chosen for additional forward analysis. As shown in Figure 13a–c, the error between dispersion curves from forwarding analysis and the theoretical solutions of the Lamb wave decreases with the reduction of measurement interval spacing. The branch of the Rayleigh wave in M_0_ is continuously disappearing when narrowing the measurement range towards the debonding region, which means that the wave is gradually spreading as a pure Lamb wave.

This study is focusing on the physical mechanism analysis of the debonding detection of rectangular CFST using mesoscale modeling approach. As illustrated in Figure 2, the frequency of the excitation signal is 250 kHz. It can be seen from Figure 10 and Figure 12 that the maximum of the concerned frequency is 500 kHz. Therefore, the sampling rate of each channel in the data acquisition system should be higher than 5 MHz/s. In this study, a total of 49 arrays of the time-history curves are observed at the same time. Accordingly, the total sampling rate of the data acquisition system should be higher than 0.245 GHz. A high-performance workstation, data acquisition system, and data transmission module are required for experimental study. It should be noted that the scientific arrangement of sensors and the relationship between the signal frequency and thickness of the steel plate should be studied in depth before performing experimental studies. In addition, the selection of the high-sensitivity sensors for the MASW measurement will be another challenging work.

## 5. Concluding Remarks

The detectability of interfacial debonding detection for rectangular CFST using the MASW method is proposed and is numerically verified in this study. The time-history propagation process of stress waves in a rectangular CFST specimen is systematically analyzed with numerical analysis modeled with the homogeneous concrete and random aggregate method. The influence of interface debonding defect on the MASW measurement and its dispersion analysis with F-K transformation is investigated in detail. The main conclusions are as follows:

(1) Due to the existence of debonding defect, the surface waves will turn into two waveforms: Lamb wave propagating in the steel plate and the diffraction wave spreading in concrete core, resulting in different distribution patterns of wave energy. The mesoscale numerical analysis indicates that the debonding defect plays a leading role in the wave propagation process of the surface wave.
(2) The specific location and dimension of the debonding defect can be identified by observing the varying tendency of the signal amplitude, wavelength, and the coaxiality characteristics of the time-history MASW measurement.
(3) For a healthy specimen, the foundation mode (M_0_) of the dispersion curves calculated with the dispersion analysis is closer to that of the theoretical curve of the Rayleigh wave. Otherwise, it will turn to be like the theoretical solutions of the Lamb wave.

Based on the surface wave theory and MASW method, the practicability of debonding detection for CFST component is investigated in this study. The research finding can provide valuable guidance on the practical application of the proposed NDT method. However, the numerical finding has to be further validated by experimental studies. Additionally, the influence of the horizontal diaphragms, vertical stiffening ribs, stud shear connectors, cross tie and the geometric shape of the CFSTs on MASW measurement should be systematically investigated. In addition, the inversion analysis of the dispersion curves and visualization of debonding defects based on shear wave velocity profiles will be performed in the follow-up studies.

## Figures and Tables

**Table 1:** Material properties of the concrete core and steel tubular of CFST.

Material	Young’s Modulus (GPa)	Poisson’s Ratio	Density (kg/m^3^)
Homogeneous concrete	32.4	0.20	2500
Aggregates	55.5	0.16	2700
Mortar	26.0	0.22	2100
ITZ	25.0	0.16	2400
Steel	207.0	0.28	7800
